# YAP and TAZ Regulate *Cc2d1b* and *Purβ* in Schwann Cells

**DOI:** 10.3389/fnmol.2019.00177

**Published:** 2019-07-17

**Authors:** Sophie Belin, Jacob Herron, Jordan J. S. VerPlank, Yungki Park, Laura M. Feltri, Yannick Poitelon

**Affiliations:** ^1^Department of Neuroscience and Experimental Therapeutics, Albany Medical College, Albany, NY, United States; ^2^Department of Cell Biology, Harvard Medical School, Boston, MA, United States; ^3^Department of Biochemistry, Hunter James Kelly Research Institute, University at Buffalo, Buffalo, NY, United States; ^4^Department of Neurology, Jacobs School of Medicine and Biomedical Sciences, University at Buffalo, Buffalo, NY, United States

**Keywords:** Yap, Taz, Cc2d1b, Purβ, myelin, Schwann cell

## Abstract

Schwann cells (SCs) are exquisitely sensitive to the elasticity of their environment and their differentiation and capacity to myelinate depend on the transduction of mechanical stimuli by YAP and TAZ. YAP/TAZ, in concert with other transcription factors, regulate several pathways including lipid and sterol biosynthesis as well as extracellular matrix receptor expressions such as integrins and G-proteins. Yet, the characterization of the signaling downstream YAP/TAZ in SCs is incomplete. Myelin sheath production by SC coincides with rapid up-regulation of numerous transcription factors. Here, we show that ablation of YAP/TAZ alters the expression of transcription regulators known to regulate SC myelin gene transcription and differentiation. Furthermore, we link YAP/TAZ to two DNA binding proteins, *Cc2d1b* and *Purβ*, which have no described roles in myelinating glial cells. We demonstrate that silencing of either *Cc2d1b* or *Purβ* limits the formation of myelin segments. These data provide a deeper insight into the myelin gene transcriptional network and the role of YAP/TAZ in myelinating glial cells.

## MAIN POINTS

YAP and TAZ regulate positive and negative myelin regulators.

Cc2d1b and Purβ are necessary for Schwann cell myelination *in vitro*.

## Introduction

The function of the nervous system relies on the ability of peripheral nerve fibers to transmit information to and from the target tissues. The speed of propagation of action potentials in these fibers is regulated by myelin, a multilamellar structure produced by Schwann cells (SCs; Monk et al., 2015). Damage to SC or peripheral myelin can be caused by numerous factors, including genetic mutations, toxic agents, inflammation, viral infections, metabolic alterations, hypoxia or physical trauma and results in severe peripheral neuropathies. SC integrate biochemical signaling pathways and mechanical stimuli coming from the extracellular matrix or from the axon (Michailov et al., 2004; Feltri and Wrabetz, 2005; Taveggia et al., 2005; Belin et al., 2017). These signals regulate an intricate network of transcription factors that control differentiation of SCs and myelination (e.g., *EGR2*, *YY1*, *ZEB2*, Topilko et al., 1994; Nagarajan et al., 2001; He et al., 2010; Weng et al., 2012; Quintes et al., 2016; Wu et al., 2016). The identification and characterization of the complete repertoire of transcription factors that modulate myelination is still incomplete (Svaren and Meijer, 2008; Fulton et al., 2011).

The identification of transcription factors responding to a specific signal is one of the first steps in dissecting the underlying regulatory networks. We showed that in *Yap* fl/+; *Taz* fl/fl; *Mpz*-Cre (*Yap* cHet; *Taz* cKO) sciatic nerves, SCs lacking YAP/TAZ are unable to myelinate and experience a global dysregulation of transcription (Poitelon et al., 2016). YAP/TAZ are two transcriptional activators of the HIPPO pathway, and play important roles in controlling organ growth, cell differentiation, proliferation and survival (Dupont, 2016). Mechanical stimulation can regulate YAP/TAZ through signals involving FAK, Src, PI3K and JNK pathways (Codelia et al., 2014; Mohseni et al., 2014; Kim and Gumbiner, 2015; Elbediwy et al., 2016), or the formation of actomyosin filaments and accumulation of F-actin (Dupont et al., 2011; Aragona et al., 2013). In addition, YAP/TAZ in SCs can be activated through Crb/Amolt proteins and laminin/G-protein signaling (Colciago et al., 2015; Fernando et al., 2016; Poitelon et al., 2016; Deng et al., 2017). YAP/TAZ regulate gene expression by binding to other DNA-binding transcription factors, especially TEAD transcription factors, but also p73, ERBB4, EGR-1 SMADs RUNXs and TBX5 (Kim et al., 2018). TEADs role in myelination is unknown (Hung et al., 2015; Lopez-Anido et al., 2015), but TEAD1 binding to transcriptional enhancers is induced during myelination (Lopez-Anido et al., 2016). Furthermore, genes encoding for essential myelin proteins (i.e., *Mpz*, *Pmp22*, *Mbp* and *Mag*) harbor TEAD elements and are downregulated in *Yap* cHet; *Taz* cKO sciatic nerves (Poitelon et al., 2016). Finally, it was suggested that YAP/TAZ and TEAD1 regulate myelin wrapping in cooperation with master myelin regulators EGR2 and SOX10 (Lopez-Anido et al., 2016; Poitelon et al., 2016).

To identify novel regulators that are essential for myelination, we integrated RNA-seq and bioinformatics analyses and looked for DNA binding proteins dysregulated in *Yap* cHet; *Taz* cKO sciatic nerves at 3 days of age. We identified two highly expressed proteins, i.e., *Cc2d1b* and *Purβ*, downregulated in *Yap* cHet; *Taz* cKO sciatic nerves. We found that silencing of *Cc2d1b* or *Purβ* *in vitro* significantly decreased the number of myelin segments and silencing of *Purβ* also significantly decreased the length of myelin segments, independently for effect on SC number, proliferation or apoptosis. These data demonstrate that CC2D1B and PURβ are necessary for myelination *in vitro*.

## Materials and Methods

### Cell Culture

Primary rat SCs were produced as described (Poitelon and Feltri, 2018) and grown with DMEM supplemented with 4 g/l glucose, 2 mM L-glutamine, 5% bovine growth serum, 2 μM forskolin, 50 ng/ml nerve growth factor, penicillin and streptomycin. SCs were not used beyond the fourth passage. Rat dorsal root ganglia (DRG) neurons from Sprague–Dawley rats embryos were isolated at embryonic day 14.5 embryos. DRG were dissociated by treatment with 0.25% trypsin and mechanical trituration and 1.5 DRGs were seeded on collagen-coated glass coverslips as described (Poitelon and Feltri, 2018). DRGs cultures were then cycled with fluoroxidine (FUDR, Sigma-Aldrich) to eliminate all non-neuronal cells. Once all non-neuronal cell remove, rat SCs were added (200,000 cells per coverslip) to establish myelinating cocultures of DRG neurons, and myelination was initiated by supplementing the medium with 50 μg/ml ascorbic acid (Sigma-Aldrich). For verteporfin (Sigma SML0534) treatment, verteporfin was solubilized in DMSO at 20 mM, then SCs were treated with either 0.5% of DMSO; 2 or 10 μM of verteporfin for 24 h. mRNA was extracted and cDNA was analyzed by RT-qPCR, as described in Poitelon et al. (2016). This study was carried out in accordance with the principles of the Basel Declaration and recommendations of ARRIVE guidelines issued by the NC3Rs and approved by the Albany Medical College Institutional Animal Care and Use Committee (no. 17-08002).

### shRNA Lentivirus Production and Infection

shRNA virions were produced as Poitelon et al. (2015). shRNA targeting *Cc2d1b* (#1, TTGCGCTCATCCCCACTGG), (#2, ATGAGCTCGAATAGCATCC) and *Purβ* (#1, AACTCGATGAGGCCCTGCG), (#2, TGGCATTGCGGTAGGATGG) and control (non-targeting) were bought from Dharmacon SMARTvector library. SCs were infected with five virions per cells, incubated for 72 h and collected for qRT-PCR analysis. Coculture experiments were done with sh*Cc2d1b* #1 and sh*Purβ* #2.

### RNA Preparation and Quantitative RT-PCR

Sciatic nerves were dissected, stripped of epineurium, frozen in liquid nitrogen, pulverized and processed as described (Poitelon et al., 2012). Total RNA was prepared from sciatic nerve or SCs with TRIzol (Roche Diagnostic). One microgram of RNA was reverse transcribed using Superscript III (Invitrogen, Carlsbad, CA, USA). For each reaction, 5 μM of oligo(dT)20 and 5 ng/μl random hexamers were used. Quantitative PCR was performed using the 20 ng of cDNA combined with 1× FastStart Universal Probe Master (Roche Diagnostic). Data were analyzed using the threshold cycle (Ct) and 2(−ΔΔCt) method. *Actb* was used as endogenous gene of reference and 18S was used as to validate the stable expression of *Actb*. The primers and probe used are the following: *18S* (F: ctcaacacgggaaacctcac, R: cgctccaccaactaagaacg, probe #77), mouse *Actb* (F: aaggccaaccgtgaaaagat, R: gtggtacgaccagaggcatac, probe #56), mouse Cc2d1b (F: cactcacaggggaaacagc, R: ctgctgccagcttctcaat, probe #4); mouse Purβ (F: aattatggctaattcggctgtt, R: tttgcagatagtcaagttttaaggttt, probe #71); rat Cc2d1b (F: gcactcactggggaaacag, R: ctgccaacttctcaatgtgg, probe #4); rat Purβ (F: aaggaactgccagcaacct, R: agactcttgcgcaggtgag, probe #56).

### Immunofluorescence and Immunoblotting

Immunohistochemistry, immunocytochemistry and immunoblotting were performed as described (Della-Flora Nunes et al., 2017). Ten microgram of protein was used for western blot. The antibodies used are the following: anti-CC2D1A (Abcam, ab68302), anti-CC2D1B (Proteintech, 20774-1-AP), anti-PURα (Proteintech, 17733-1-AP), anti-PURβ (Proteintech, 18128-1-AP), anti-calnexin (Sigma, C4731), anti-phospho-histone H3 (Millipore, 06-576), anti-neurofilament H (Biolegend, 822701), anti-MBP (Biolegend, 808401). CC2D1B antibody was validated using *Cc2d1b* knockout mouse (Zamarbide et al., 2018). PURβ antibody was validated using purified PURβ fusion protein (Proteintech, Ag12705). Briefly, 1.5 μg of PURβ antibody was incubated with 50 μg of Purβ protein for 1 h at 37°C prior to being used for immunohistochemistry. TUNEL assays were performed on coverslips of culture as described in Poitelon et al. (2016). Myelination *in vitro* was evaluated from three different experiments, performed with two coverslips in each case, which is a standard sample size for these experiments. Images were acquired with identical acquisition parameters on an epi fluorescent Axio Imager A2 (Zeiss). Myelin segments number and length were quantified using ImageJ software^1^ from two random fields of each culture at the 10× objective, as described in Ghidinelli et al. (2017).

### Bioinformatics

RNAseq data were obtained from NCBI GEO: GSE79115 (Poitelon et al., 2016). Genes encoding for DNA-binding protein genes were predicted thanks to transcriptionfactor.org database. The expression data for *Cc2d1b* and *Purβ* were obtained from mousebrain.org/ and gtexportal.org on 09/2018.

### Statistical Analyses

Experiments were not randomized, but data collection and analysis were performed blind to the conditions of the experiments. Data excluded are reported in the legend of the figures. Data are presented as mean ± standard error of the mean (SEM) or SD. No statistical methods were used to predetermine sample sizes, but our sample sizes are similar to those generally employed in the field. Two-tailed Student’s *t*-test, One-way analysis of variance (ANOVA) and Two-way ANOVA were used for statistical analysis of the differences between multiple groups according to the number of sample groups. Values of *P* ≤ 0.05 were considered to represent a significant difference.

## Results

### YAP and TAZ Regulate DNA-Binding Proteins in Schwann Cells

**Figure 1:**
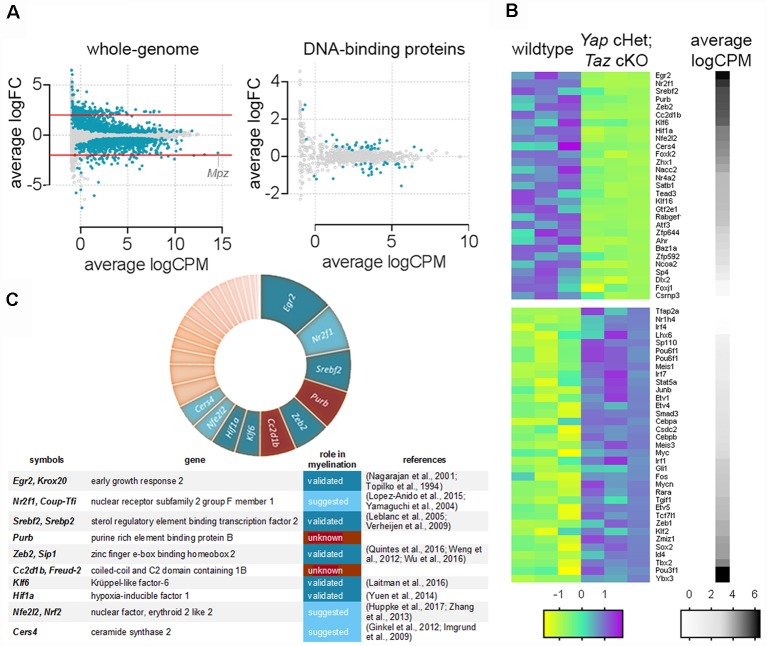
Genes encoding DNA-binding proteins significantly repressed in *Yap* cHet; *Taz* cKO sciatic nerves at 3 days of age. **(A)** Scatter plot for the comparison between genes differentially expressed in the wildtype and *Yap* cHet; *Taz* cKO sciatic nerves at 3 days of age. Log_2_ fold-change in *Yap* cHet; *Taz* cKO; vs. control mice was plotted against the average count size (log-counts-per-million) for every gene. Blue dots indicate statistically different genes (False Discovery Rate ≤0.05). The x-axis (logCPM, log counts per million) is a measure of gene expression, with higher numbers indicating genes highly expressed in sciatic nerves (e.g., *Mpz*). The y-axis (logFC, log base 2-fold change) indicates if ablation of *Yap*/*Taz* upregulate or downregulate gene expression. Genes with positive values on the y-axis are positively regulated in *Yap* cHet; *Taz* cKO sciatic nerves when compared to control, while those with negative values on the y-axis are negatively regulated in *Yap* cHet; *Taz* cKO sciatic nerves. Red lines indicate a 2-fold difference. On the left, expression levels of all genes expressed in sciatic nerves are represented (whole-genome). Among these, 2,071 out of 18,016 genes are dysregulated (Poitelon et al., 2016). On the right, genes encoding for a DNA-binding protein were selected for the presence of a DNA binding domain (http://www.transcriptionfactor.org) expression levels of all DNA-binding proteins expressed in sciatic nerves are represented. Among these, 64 out of 1,445 are dysregulated. Average logCPM were calculated as log_2_ (average CPM + 0.5). **(B)** Heatmap for the significantly dysregulated DNA-binding proteins in *Yap* cHet; *Taz* cKO sciatic nerves at 3 days of age. The differential expression of genes encoding for a DNA binding domain protein was tested in wildtype and *Yap* cHet; *Taz* cKO sciatic nerves. Of the 1,445 genes tested, 64 showed statistical significance. Colors in this heatmap correspond to expression levels in *Yap* cHet; *Taz* cKO vs. control sciatic nerves, on a scale of yellow for lowest values to magenta for highest values with cyan for moderate values. Genes are categorized based on their expression levels in wildtype sciatic nerves (black denotes high expression levels, whereas white depicts low expression levels). Heatmap data are calculated using *Z*-score, where *z* = x-mean in the samples/standard deviation in the samples. **(C)** The chart categorized DNA-binding proteins repressed *Yap* cHet; *Taz* cKO sciatic nerves according to their expression levels in wildtype sciatic nerves (segment width indicates expression level). Among the 10 most expressed DNA-binding proteins (*Egr2*, *Nr2f1*, *Srebf2*, *Purβ*, *Zeb2*, *Cc2d1b*, *Klf6*, *Hif1a*, *Nfe2l2*, *Cers4*), eight have a role to play in myelin formation (blue). The role of *Purβ* and *Cc2d1b* in myelination is unknown (brown).

To examine the function of YAP and TAZ at the whole-genome level we analyzed RNA-seq transcriptome profiling of *Yap* cHet; *Taz* cKO sciatic nerves at 3 days of age (NCBI GEO: GSE79115). We identified 2,071 misregulated transcripts (Figure 1, Poitelon et al., 2016). We narrowed our analysis to DNA-binding proteins and identified that ablation of *Yap*/*Taz* dysregulated 64 genes (Figure 1B). Genes encoding for DNA-binding proteins were then categorized according to their level of expression (Figure 1B, black/white heatmap). Signature genes normally expressed in neural crest cells (*Tbx2*) and immature SCs (*Oct6/Pou3f1*/*Scip*), as well as genes inhibiting differentiation (*Id4*) and myelin formation (*Sox2*) were highly expressed (Figure 1B, black) and upregulated in *Yap* cHet; *Taz* cKO sciatic nerves (Figure 1B, magenta; Arroyo et al., 1998; Jang et al., 2010; Ma et al., 2015; Roberts et al., 2017). Genes involved in myelination were highly expressed and downregulated (Figure 1C). Among the 10 most expressed DNA-binding proteins that were downregulated in *Yap* cHet; *Taz* cKO sciatic nerves, eight were already shown or suggested to play a role in myelination: *Egr2*, *Nr2f1*, *Srebf2*, *Zeb2*, *Klf6*, *Hif1a*, *Nfe2l2* and *Cers4* (Figure 1C; Topilko et al., 1994; Nagarajan et al., 2001; Yamaguchi et al., 2004; Leblanc et al., 2005; Imgrund et al., 2009; Verheijen et al., 2009; Ginkel et al., 2012; Weng et al., 2012; Zhang et al., 2013; Yuen et al., 2014; Lopez-Anido et al., 2015; Laitman et al., 2016; Quintes et al., 2016; Wu et al., 2016; Huppke et al., 2017). Excitingly, the remaining two DNA-binding proteins *Cc2d1b* and *Purβ*, have no known roles in peripheral nervous system development or myelination.

### Identification of Novel Myelin Regulators in Schwann Cells

*Cc2d1b*, also named *Freud-2*, encodes for Coiled-coil and c2 domain containing 1B protein and is highly expressed in peripheral nerves and myelinating oligodendrocytes^2^^,^^3^ (Zhang et al., 2014). *Purβ* encodes for the Purine Rich elem//ent binding protein B. *Purβ* binding elements have already been characterized in numerous genes, including *Mbp* and *Plp1* (Tretiakova et al., 1999; Dobretsova et al., 2008), yet it is unclear if *Purβ* is necessary for their expression.

**Figure 2:**
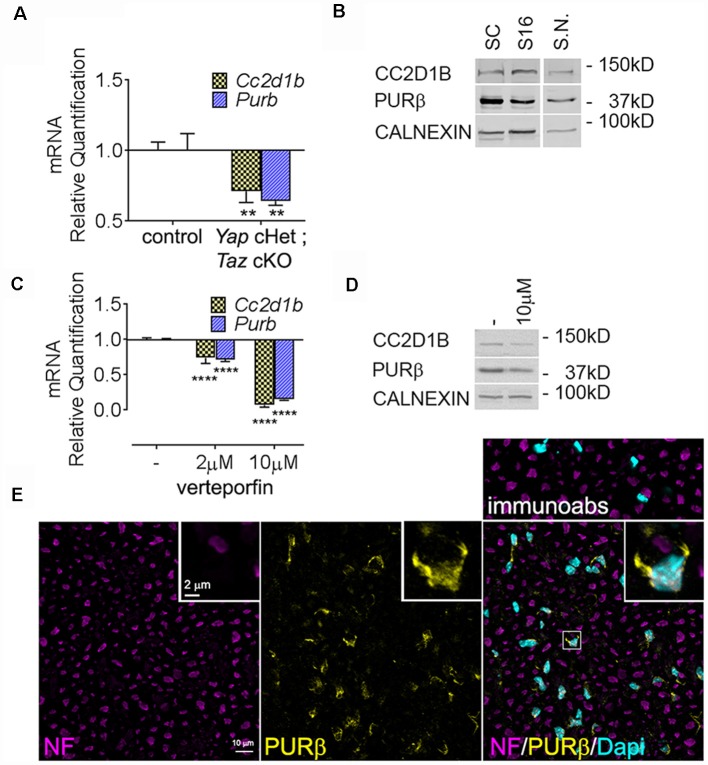
Expression of *Cc2d1b* and *Purβ* in sciatic nerves and SCs. **(A)** Quantitative RT-PCR analysis confirmed a transcriptional repression of *Cc2d1b* (−29%) and *Purβ* (−36%) in *Yap* cHet; *Taz* cKO sciatic nerves at P3. *n* = 3 animals per genotype. Two-way analysis of variance (ANOVA) with Bonferroni *post hoc* test. *F*_(1,8)_ = 49.64, *P* = 0.0001, *Cc2d1b* *P* = 0.0044, *Purβ* *P* = 0.0011. Error bars indicate standard error of the mean (SEM). **(B)** Western blot analysis using the rabbit anti- CC2D1B and PURβ antibodies on wildtype rat SCs (lane 1), S16 SCs (lane 2) and wildtype mouse sciatic nerves (S.N.) at 30 days of age (lane 3). Calnexin was used as loading control. **(C)** mRNA and **(D)** protein levels of *Cc2d1b* and *Purβ* after verteporfin treatment (relative to DMSO-treated controls) in SCs. *Cc2d1b* (−93%) and *Purβ* (−85%) mRNA are strongly reduced upon a 24 h treatment with 10 μM of verteporfin. CC2D1B (−44%) and PURβ (−38%) protein levels are also reduced after a 24 h treatment with 10 μM of verteporfin. *n* = 3 independent experiments with three independent samples per group for **(A,C)**. Error bars indicate SD. A logarithmic scale was used for the *y*-axis of **(A,C)**, and the origin was set to 1. Two-way ANOVA with Bonferroni *post hoc* test. *F*_(2,12)_ = 817.9, *P* < 0.0001, *Cc2d1b* *P* < 0.0001, *Purβ* *P* < 0.0001. **(E)** Immunolocalization of PURβ in cross section of sciatic nerve at P30 show localization of PURβ in SC cytoplasm and nuclei, although at P30 PURβ staining appears to be more intense in SC cytosol than in the nuclei. Scale bar = 10 μm. Insert display a magnified SCs stained for PURβ. Insert scale bar = 2 μm. Immunoabs displays a validation for PURβ antibody for which PURβ antibody was pre-absorbed with 30-fold excess of the PURβ protein before immunohistochemistry. **P < 0.01, ****P < 0.0001.

We first confirmed our RNAseq data by qRT-PCR and showed that *Cc2d1b* and *Purβ* are downregulated in *Yap* cHet; *Taz* cKO sciatic nerves (Figure 2A). Because dysregulation of gene expression in sciatic nerves can be due to alterations of mRNA level in SCs, axons, perineurial or endothelial cells or fibroblasts, we confirmed that *Cc2d1b* and *Purβ* are expressed by primary SCs (Figures 2B,E). Finally, we showed that treatment of primary rat SCs with verteporfin, a drug that inhibits YAP/TAZ regulation of transcription by disrupting its interactions with TEAD transcription factors, reduces expression of *Cc2d1b* and *Purβ* (Figures 2C,D). Altogether, these data indicate that CC2D1B and PURβ are expressed by SCs and regulated by YAP/TAZ/TEAD.

### *Cc2d1b* and *Purβ* Regulate Myelination *in vitro*

**Figure 3:**
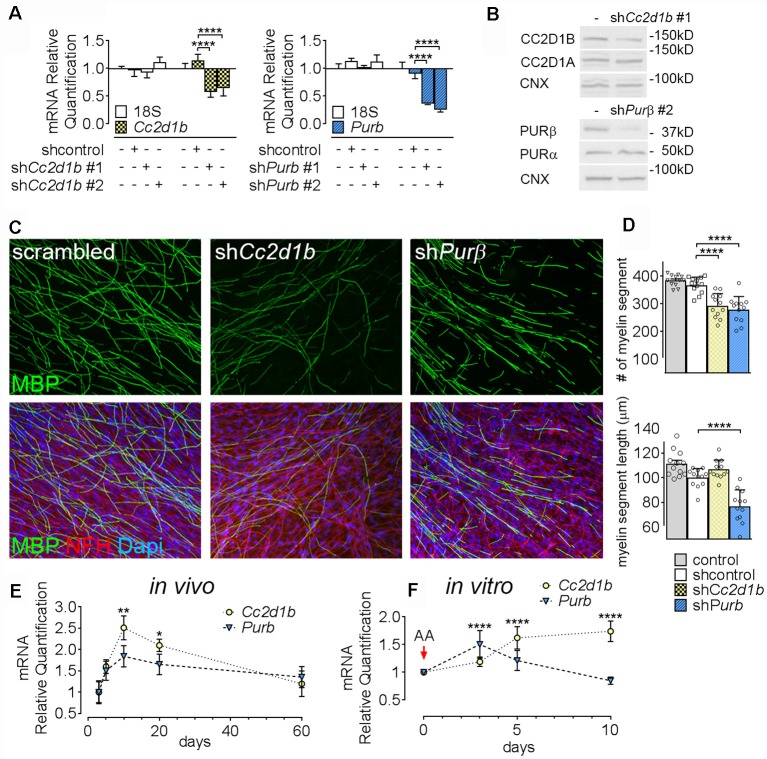
*Cc2d1b* and *Purβ* regulate myelination *in vitro*. **(A)** Quantitative RT-PCR analysis showed the silencing efficiency of shRNAs targeting *Cc2d1b* and *Purβ* in primary rat SCs. *n* = 3 experiments. One-way ANOVA with Bonferroni *post hoc* test. *Cc2d1b* *F*_(3,16)_ = 10.83, *P* = 0.0004, sh*Cc2d1b* #1 (−43%) *P* < 0.0001 sh*Cc2d1b* #2 (−36%) *P* < 0.0001, *Purβ* *F*_(3,16)_ = 35.09, *P* < 0.0001, sh*Purβ* #1 (−64%) *P* < 0.0001, sh*Purβ* #2 (−75%) *P* < 0.0001. Error bars indicate SD. A logarithmic scale was used for the y-axis and the origin was set to 1. **(B)** Western analysis showed that silencing *Cc2d1b* and *Purβ* reduce CC2D1B (−41%) and PURβ (−80%) protein levels, but do not after their respective homologs (CC2D1A and PURα). **(C,D)** Immunolocalization of myelin protein revealed a defect in myelin production in SCs silenced for either *Cc2d1b* or *Purβ*. SCs (200,000 cells) infected with shRNA were seeded on dorsal root ganglia (DRG) neurons and allowed to myelinate for 10 days. Cultures were stained for Myelin Basic Protein (MBP, green), neurofilament H (NFH, red) and DAPI (blue). The number and length of myelin segments were quantified. A lower number of segments were observed in both sh*Cc2d1b* and sh*Purβ* and shortened myelin segments were observed in sh*Purβ*. *n* = 4 coverslips for three independent experiments. One-way ANOVA with Bonferroni *post hoc* test. #myelin segments *F*_(3,44)_ = 24.31, *P* < 0.0001, sh*Cc2d1b* *P* < 0.0001, sh*Purβ* *P* < 0.0001. myelin segments length *F*_(3,44)_ = 28.03, *P* < 0.0001, sh*Purβ* *P* < 0.0001. Error bars indicate SD. **(E)** Quantitative RT-PCR analysis demonstrated a transient upregulation of *Cc2d1b* mRNA (dotted line, yellow circles) in sciatic nerves during *in vivo* myelination, from 10 to 20 days of age. *Purβ* expression (dashed line, blue triangles) also tend to be increased at 10 days of age. *n* = 3 animals. One-way ANOVA with Bonferroni *post hoc* test. *F*_(4,20)_ = 7.51, *P* = 0.0007, *Cc2d1b* 10 days *P* = 0.001, *Cc2d1b* 20 days *P* = 0.017, *Purβ* 10 days *P* = 0.09. Error bars indicate SEM. **(F)** Quantitative RT-PCR analysis demonstrated an upregulation of *Cc2d1b* (dotted line, yellow circles) in SC-neuron cocultures during *in vitro* myelination, from 5 days after addition of ascorbic acid (AA). *Purβ* (dashed line, blue triangles) is also transiently upregulated, 3 days after addition of AA. *n* = 6 coverslips. One-way ANOVA with Bonferroni *post hoc* test. *F*_(3,40)_ = 17.26, *P* < 0.0001, *Cc2d1b* 5 days *P* < 0.0001, *Cc2d1b* 10 days *P* < 0.0001, *Purβ* 3 days *P* < 0.0001, *Purβ* 5 days *P* = 0.05. Error bars indicate SD. ****P < 0.0001, ***P < 0.001, **P < 0.01, *P < 0.05.

**Figure 4:**
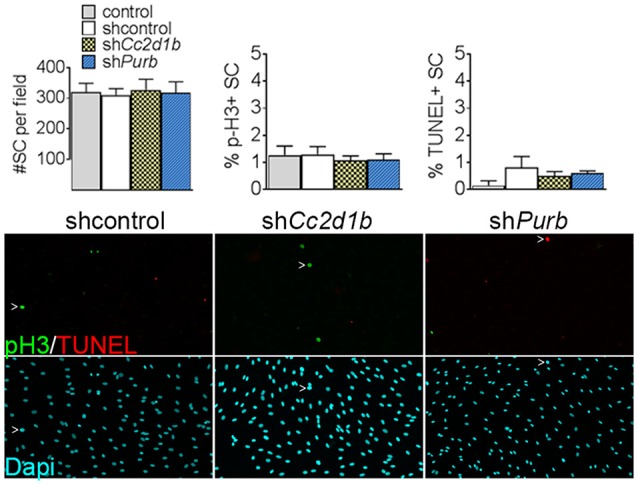
*Cc2d1b* and *Purβ* do not increase SC proliferation or apoptosis. Dapi (blue) staining on SC silenced for either *Cc2d1b* and *Purβ* show no differences in cell number. TUNEL analysis (red) and phospho-Histone 3 (p-H3—green) staining show no increase in proliferation or apoptosis. SCs were infected with five virions per cells, incubated for 72 h and stained. *n* = 4 independent experiment. One-way ANOVA with Bonferroni *post hoc* test. Error bars indicate SD.

To determine the function of CC2D1B and PURβ in SCs, we asked if silencing the expression of *Cc2d1b* and *Purβ* in SCs would affect the capability of SC to myelinate axons. SCs were infected with viruses expressing different shRNAs for either *Cc2d1b* or *Purβ*. All shRNAs reduced expression of *Cc2d1b* or *Purβ*, as shown by quantitative RT-PCR and western blot (Figures 3A,B). In addition, we show that silencing *Cc2d1b* or *Purβ* does not affect the protein level of their homolog CC2D1A and PURα (Figure 3B). When SCs silenced for *Cc2d1b* and *Purβ* were seeded on DRG neurons and cocultured in myelinating conditions by adding ascorbic acid to the cultures, myelination of axons was impaired (Figures 3C,D). Because a defect of myelination can be caused by a reduced number of SCs attached to axons, we asked whether silencing Cc2d1b or Purβ caused changes in apoptosis or proliferation. However, silencing of *Cc2d1b* and *Purβ* did not affect SC proliferation or apoptosis (Figure 4). Finally, during *in vivo* and *in vitro* myelination, *Mbp* and *Mpz* gene expression peak 3 days after birth and 40 days after addition of ascorbic acid, respectively (Notterpek et al., 1999). We showed that *Purβ* expression was not significantly altered during SC development (Figure 3E) but appears, *in vitro*, to spike after 3 days in culture, before the start of myelination (Figure 3F). In contrast, *Cc2d1b* was highly expressed *in vivo* between postnatal day 5 (P5) and P20 (Figure 3E), and *in vitro* after 5 days in culture (Figure 3F), when SCs myelinate axons. Taken together, these data show that CC2D1B or PURβ is required for SC myelination *in vitro*.

## Discussion

In this study, we identify novel regulators essential for myelination. *Yap* cHet; *Taz* cKO sciatic nerves present an arrest SC early development and an abolition of subsequent SC myelination. We first hypothesized that genes regulated by YAP/TAZ include novel regulators of myelin formation. Thus, we analyzed gene expression by RNA-Seq analysis in *Yap* cHet; *Taz* cKO sciatic nerves. In contrast to classical analyses based on gene dysregulation, which would highlight genes highly regulated by YAP/TAZ (Poitelon et al., 2016), we used our dataset to look at DNA binding proteins highly expressed in *Yap* cHet; *Taz* cKO sciatic nerves, with the secondary assumption that their level of expression would be correlated to their importance in myelin formation. In this report, we validate our hypotheses and found that global gene expression stratification allows for the identification of genes essential for myelination. We were able to identify that most of the genes known to be either activators or inhibitors of myelination are highly expressed in sciatic nerves and are dysregulated in *Yap* cHet; *Taz* cKO. Following our reasoning, we identify two novel DNA binding proteins CC2D1B and PURβ, with previously unsuspected role in myelinating glial cells. CC2D1B and PURβ are highly expressed in sciatic nerves and in SCs and downregulated in *Yap* cHet; *Taz* cKO. We demonstrate that CC2D1B and PURβ are required for normal myelination. Ablation of either CC2D1B or PURβ impairs myelination *in vitro*, independently from effects on SC proliferation or apoptosis. Altogether our data demonstrate that CC2D1B and PURβ are both involved in myelin formation *in vitro*.

CC2D1B and PURβ are both DNA-binding proteins, but their role as a regulator in myelinating glial cells or other cells remains undefined. CC2D1B protein structure is close to its homolog CC2D1A.

*Cc2d1a* is highly expressed in neurons and has been implicated in intellectual disability and autism spectrum disorder (Basel-Vanagaite et al., 2006; Manzini et al., 2014), *Cc2d1b* is expressed in myelinating glial cells and peripheral nerves^4^^,^^5^ (Zhang et al., 2014), which indicates that its role and function might not be fully redundant with *Cc2d1a*.

Few studies have suggested a redundant role between both proteins for the regulation of serotonin receptors (Hadjighassem et al., 2009, 2011). Yet, the transcriptional role of CC2D1 remains controversial, as other studies showed that both CC2D1 are confined to the cytoplasm and perinuclear endosomes (Drusenheimer et al., 2015). Thus, it remains unclear whether CC2D1B can directly control transcription *in vivo* and whether it translocates from the cytoplasm to the nucleus. Interestingly, other functions have been proposed for CC2D1 proteins independently from their DNA-binding domain. CC2D1 proteins belong to the evolutionary conserved Lgd protein family which was shown to be involved in the regulation of signaling receptor degradation *via* the endosomal pathway (Jaekel and Klein, 2006). Loss of Lgd function results in an ectopic and ligand-independent activation of the Notch pathway (Childress et al., 2006; Gallagher and Knoblich, 2006; Jaekel and Klein, 2006). Notch signaling promotes the early stage of SC development but inhibits myelination (Woodhoo et al., 2009). Thus, it is possible that CC2D1B modulates myelination through the recruitment of specific signaling complexes. Finally, *Cc2d1b* KO mice have been reported and present delayed memory acquisition and retention (Zamarbide et al., 2018). There is growing evidence, both from animal studies and human neuroimaging that myelin plays a role in learning (McKenzie et al., 2014; Sampaio-Baptista and Johansen-Berg, 2017) and it might be worthwhile to also consider the role of CC2D1B in central nervous system myelination.

PURβ belongs to the purine-rich element binding (PUR) protein family, which includes of PURα, PURβ and PURγ. There is substantial evidence for PUR role in DNA binding (Rumora et al., 2013; Ferris and Kelm, 2019). Among them, PURα was studied the most, for its implication in fragile × syndrome and PURA syndrome, a disorder characterized by intellectual disability and delayed development of speech and motor skills, such as walking (Johnson et al., 2013; Hunt et al., 2014; Lalani et al., 2014). Interestingly, *Pur*α knockout mice present with reduced myelin production and pathologic development of glial cells (Khalili et al., 2003). However, no knockouts of the other PUR family genes have been reported. PURβ is known to play a role in cell differentiation and modulates transcriptional regulation of gene expression of the α-and β-myosin heavy chain and actin α-2 (Gupta et al., 2003; Ramsey and Kelm, 2009). Notably, TEAD-1, the main transcriptional partner of YAP/TAZ, was reported to be upstream of *Purβ* (Tsika et al., 2010). TEAD motif-harboring enhancers (GGAAT) can be found in numerous genes dysregulated in *Yap* cHet; *Taz* cKO, including CC2D1B and PURβ. Yet, no binding motifs for EGR2 or SOX10 were found in the PURβ enhancer region (Heinz et al., 2010). Thus, it is possible that PURβ is a promyelinating regulator directly downstream of YAP/TAZ/TEAD1. Finally, all PUR isoforms have been associated with neoplasia (Johnson et al., 2013).

Although the *Cc2d1b* and *Purβ* expression appear to be downstream YAP/TAZ/TEADs, the signals contributing to the regulation of their expression are essentially unknown. Elucidation of the upstream pathways and signals that induce CC2D1B and PURβ will be important both for understanding the molecular control of the myelination program, but also potentially for identifying strategies to promote remyelination in demyelinating disease. Indeed, numerous transcription factor involved in developmental myelination is also involved in remyelination following injury. Thus, it will be critical to characterize the role of CC2D1B and PURB in peripheral nerve repair.

Finally, our study extends regulatory mechanisms directing SC myelinogenesis (Hung et al., 2015; Boerboom et al., 2017; Quintes and Brinkmann, 2017) and supports the transition from a gene-centric to a network-systems view of the myelin formation. Further characterization of the transcription factor network controlling myelin gene expression should help refine our understanding of SC development as well as suggest novel therapeutic strategies to potentiate their regenerative capacity.

## Data Availability

All datasets generated for this study are included in the manuscript and/or the supplementary files.

## Author Contributions

SB and YPo designed research and interpreted data. SB and YPo performed experiments with JH assistance. YPa contributed to analytical tools. YPo wrote the manuscript with SB assistance. SB and YPo analyzed the data. YPa, JV, and LF critically reviewed the manuscript.

## Conflict of Interest Statement

The authors declare that the research was conducted in the absence of any commercial or financial relationships that could be construed as a potential conflict of interest.

## Footnotes

^1^http://imagej.nih.gov/ij

^2^www.mousebrain.org

^3^www.gtexportal.org

^4^www.mousebrain.org

^5^www.gtexportal.org
