# Corrigendum: YAP and TAZ Regulate *Cc2d1b* and *Pur*β in Schwann Cells

**DOI:** 10.3389/fnmol.2019.00256

**Published:** 2019-10-18

**Authors:** Sophie Belin, Jacob Herron, Jordan J. S. VerPlank, Yungki Park, Laura M. Feltri, Yannick Poitelon

**Affiliations:** ^1^Department of Neuroscience and Experimental Therapeutics, Albany Medical College, Albany, NY, United States; ^2^Department of Cell Biology, Harvard Medical School, Boston, MA, United States; ^3^Department of Biochemistry, Hunter James Kelly Research Institute, University at Buffalo, Buffalo, NY, United States; ^4^Department of Neurology, Jacobs School of Medicine and Biomedical Sciences, University at Buffalo, Buffalo, NY, United States

**Keywords:** Yap, Taz, Cc2d1b, Purβ, myelin, Schwann cell

In the published article, there was a mistake in all author names. The first and last names of all authors were mistakenly inverted. The correct author list with the correct first and last name order appears below.

“Sophie Belin^1^, Jacob Herron^1^, Jordan J. S. VerPlank^2^, Yungki Park^3^, Laura M. Feltri^3, 4^, and Yannick Poitelon^1*^”

This does not change the scientific conclusions of the article in any way. The original article has been updated.

